# Introducing and Boosting Oxygen Vacancies within CoMn_2_O_4_ by Loading on Planar Clay Minerals for Efficient Peroxymonosulfate Activation

**DOI:** 10.3390/molecules29163825

**Published:** 2024-08-12

**Authors:** Xue Yang, Xiao Yao, Yinyuan Qiu

**Affiliations:** 1 School of Ecological Environment and Urban Construction, Fujian University of Technology, Fuzhou 350118, China; 19046048r@connect.polyu.hk; 2 Fujian Special Equipment Inspection and Research Institute, Fuzhou 350008, China; 3 School of Mechanical and Automotive Engineering, Fujian University of Technology, Fuzhou 350118, China

**Keywords:** composite catalyst, CoMn_2_O_4_, kaolinite, peroxymonosulfate activation, pharmaceutical degradation

## Abstract

CoMn_2_O_4_ (CMO) has been recognized as an effective peroxymonosulfate (PMS) activator; however, it still shows disadvantages such as limited reactive sites and metal leakage. Herein, an effective and environmentally friendly composite catalyst, CMO/Kln, was synthesized by anchoring CMO on kaolinite (Kln), a natural clay mineral with a special lamellar structure, to activate peroxymonosulfate (PMS) for the degradation of residue pharmaceuticals in water. The abundant hydroxyl groups located on the surface of Kln helped induce rich oxygen vacancies (OVs) into composite CMO/Kln, which not only acted as additional active sites but also accelerated working efficiency. In addition, compared with bare CMO, CMO/Kln showed lower crystallinity, and the adoption of the Kln substrate contributed to its structural stability with lower metal leaching after three rounds of reaction. The universal applicability of CMO/Kln was also verified by using three other pharmaceuticals as probes. This work shed light on the adoption of natural clay minerals in modifying CMO catalysts with promoted catalytic activity for the efficient and eco-friendly remediation of pharmaceuticals in wastewater.

## 1. Introduction

Persistent organic pollutants like pharmaceuticals can hardly be completely removed by traditional sewage treatment plants (STPs); thus, they are frequently detected in surface water at concentrations ranging from ng/L to μg/L [1]. These pharmaceutical molecules and their metabolites cause secondary pollution in the receiving water bodies, which gives rise to adverse ecological effects like aquatic toxicity, the development of resistance in pathogenic bacteria, genotoxicity, and endocrine disruption [2,3]. Therefore, sulfate radical (SO_4_^·−^)-based advanced oxidation processes (SR-AOPs) have drawn increasing attention from researchers worldwide due to their stability, efficiency, and high redox potential (2.8 V). SO_4_^·−^ can be generated from activated peroxymonosulfate (PMS) or peroxydisulfate (PDS) by various stimuli, among which transitional metal-based catalysts have been widely adopted since they are cheap and easy to operate with less complex reactors [4]. Although catalysts containing Co^2+^ have demonstrated superior catalytic activity compared with other transition metals, exposure to excessive Co can cause cobalt poisoning in the human body and adversely affect the atmosphere, water, and soil [5,6,7]. Therefore, CoMn_2_O_4_ with the spinel structure has been widely used in AOPs due to its structural stability and high catalytic efficiency [8]. During its preparation, a special redox loop with a mid-valence state, Mn^2+^/Mn^3+^/Mn^4+^, could be formed, probably providing more electron transfer pathways [9,10]. Additionally, the substitution of Mn can reduce the toxicity from the Co element [11]. However, bare CoMn_2_O_4_ still has obvious disadvantages, such as the serious aggregation during the crystallization, which retards electron transfer efficiency and the remained Co leaching, which interferes with its catalytic activity [12].

One of the solutions to mitigate the above-mentioned issues is to induce substrates like metal–organic frameworks (MOFs) and carbon or titanite materials for the in situ growth of CoMn_2_O_4_ nanoparticles [13,14,15,16]. However, most of the mentioned materials suffer from defects such as costly, time-consuming, and complex preparation [17]. Conversely, natural clay minerals with characteristics like porosity, chemical stability, abundance, large surface area, and eco-friendliness have gradually received attention in environmental remediation, especially in air and water purifications [18,19]. A larger specific area could help improve the dispersion of nanoparticles, and the abundant hydroxyl groups on the mineral’s surface could anchor nanoparticles to enhance its stability and PMS activation ability [19].

Kaolinite (Kln) is a typical phyllosilicate mineral belonging to the kaolin subgroup, which shares a general composition expressed as Al_2_Si_2_O_5_(OH)_4_·nH_2_O. The lamellar Kln is composed of dioctahedral 1:1 layers linked through -Al-O-H...O-Si- hydrogen bonding [20,21]. The layers of Kln are essentially neutral, and its abundant aluminum hydroxyl groups can effectively prevent self-agglomeration of the carried nanoparticles [22].

In this work, bare CoMn_2_O_4_ (CMO) nanocatalyst and complex ones anchoring CMO on Kln were synthesized separately, and their PMS activation ability was evaluated by removing sulfamethoxazole (SMZ), a frequently detected antibiotic, from water. The morphology, crystal phase, and pore structures of the catalysts were analyzed. The goals of this work were as follows: (1) determining the optimal working parameters for the catalysts; (2) analyzing the PMS activation mechanisms and dominant ROS within the reaction system; (3) investigating the recyclability, reusability, and stability of these catalysts; (4) evaluating the general applicability of the catalysts by testing on some other pharmaceuticals; and (5) proposing the critical roles of the Kln substrate. The study aimed to shed light on the adoption of natural minerals in fabricating catalysts for PMS activation and provide a fundamental understanding of different degradation performances caused by a mineral carrier’s structure difference.

## 2. Results and Discussion

### 2.1. Structural and Physicochemical Properties of the Catalysts

**Figure 1:**
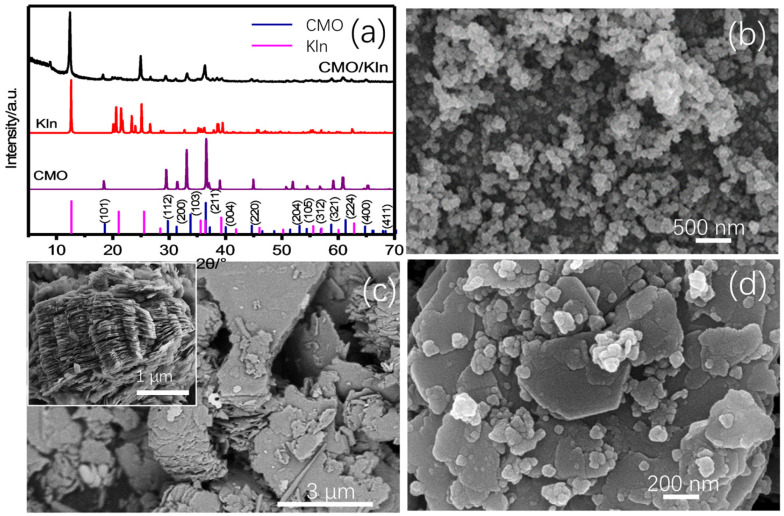
XRD patterns of bare CMO, Kln, and CMO/Kln (**a**), and FESEM images of bare CMO, Kln, and CMO/Kln (**b**–**d**).

The XRD patterns in Figure 1a show that both bare CMO and Kln corresponded well with their standard cards, the cubic cobalt dimanganese (III) oxide (CoMn_2_O_4_, JCPDS No. 77-0471) and kaolinite (JCPDS No. 14-0164). The CMO/Kln series presented a clear two-phase composition of both CMO and Kln minerals without other reflection peaks, revealing the successful anchoring of CMO to Kln with high purity. The presence of Kln significantly reduced the intensity of the sharp CMO signals, and some widened peaks were observed occasionally, which implied the weakened crystallinity of CMO/Kln compared with bare CMO. The slight shifts of CMO/Kln compared with bare Kln toward a higher angle were caused by the lattice changes in Kln due to the integration of CMO. The average grain sizes of bare CMO and CMO loaded on Kln were calculated using the Scherrer formula separately, considering their most intense peaks, at about 26 and 12 nm (detailed in Supporting Information, Text S1). Thus, the induction of the Kln substrate also helped reduce the grain size of CMO.

The FESEM images in Figure 1b show that without the Kln substrate, the CMO particles seriously aggregated and tended to form into cubic crystals as bulk crystals were likely to be formed through homogeneous co-precipitation [23]. Bare Kln appeared with a regular lamellar structure (Figure 1c), and the attachment of CMO induced little changes in its morphology (Figure 1d). The fine CMO particles spread across the surfaces of Kln evenly with obviously alleviated agglomeration. Clustered CMOs were occasionally found on Kln’s nanosheets but hardly on the surrounding sides. 

**Figure 2:**
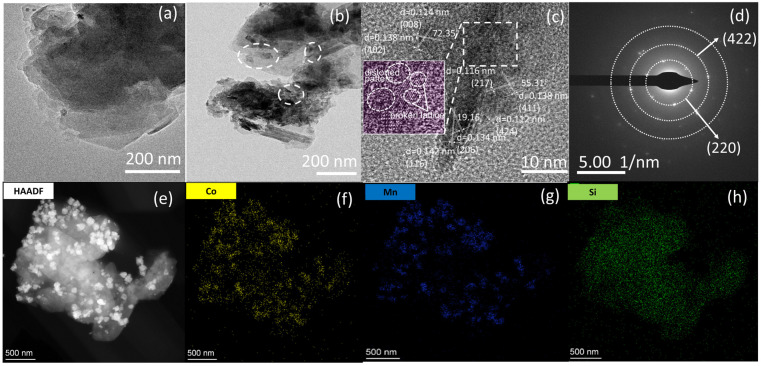
STEM images of raw Kln (**a**) and CMO/Kln (**b**,**c**); SAED pattern of CMO/Kln (**d**); and EDS mappings of CMO/Kln (**e**–**h**).

The distribution pattern of CMO particles on the Kln substrate’s nanosheet was also observed clearly through HAADF-STEM. The stacking kaolinite nanosheets with smooth and well-defined edges remained almost unchanged after the loading of CMO particles, and the spinel aggregates were distributed evenly on the surface of Kln (Figure 2a,b). Subtle features of CMO/Kln were further investigated by analyzing its lattice fringes. As labeled in Figure 2c, the lattice fringes of CMO/Kln at 0.114, 0.138, and 0.142 nm are well indexed to the interplanar spacing of cubic CMO—namely, (008), (402), and (116), respectively—indicating the multidirectional crystallization of CMO on Kln. Furthermore, several overlaps and blurred boundaries were found in the lattice fringes of CMO/Kln, which might indicate the decreased particle size of CMO with lower crystallinity. This is consistent with CMO/Kln’s XRD pattern with weaker diffraction peaks in Figure 1a. Moreover, the lattice fringes with overlaps, crossings, and blurred lattice borders are commonly considered promoters for catalytic ability since they provide more active sites and tend to have more lattice defects [24,25]. Thus, it could be inferred that the Kln substrate helped disperse CMO particles evenly and suppress their long-range ordering in unidirectional crystallization [26]. The SAED pattern of CMO/Kln in Figure 2d shows several bright random dots and a series of four-fold symmetric diffraction rings, which look slightly thick with occasional breaks. This implies the polycrystalline state of CMO/Kln, which is consistent with its XRD patterns and lattice fringes previously obtained from HRTEM [27]. The lattice planes of the SAED rings calculated from the interlayer spacing agreed well with CMO, which further revealed the multidirectional crystallization and successful attachment of CMO to Kln. The EDS mappings of CMO/Kln in Figure 2e–h verify the homogeneous dispersion of Co, Mn, and Si elements, which also confirms the successful combination and uniform distribution of CMO particles on the Kln substrate.

**Figure 3:**
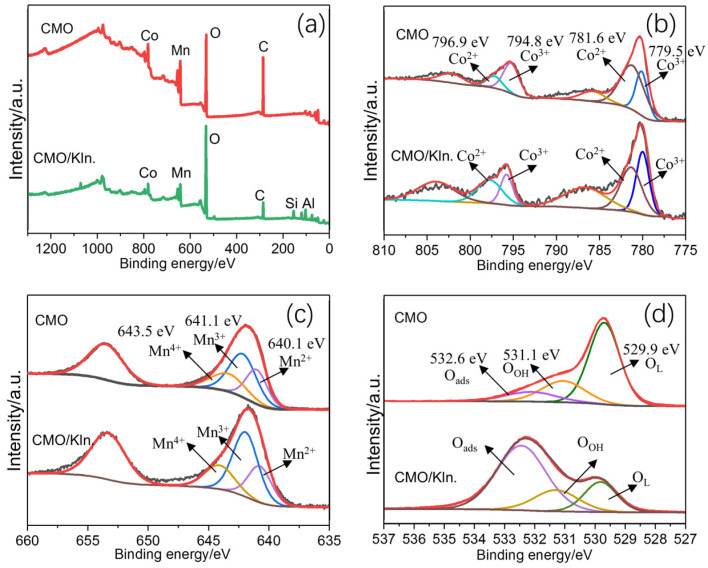
XPS spectra of survey (**a**), Co 2p (**b**), Mn 2p (**c**), and O 1s (**d**) for bare CMO and CMO/Kln.

The XPS survey spectra in Figure 3a confirm the existence of Co, Mn, and O in bare CMO, while Al and Si are additionally found in CMO/Kln. The atomic surface ratio of Co/Mn within CMO/Kln obtained here was 0.48, close to the 1:2 feeding ratio during the synthesis. This indicated the successful fabrication of the catalysts. 

The Co 2p spectra in Figure 3b of the catalysts can be divided into two major peaks, Co 2p 3/2 and Co 2p 1/2, at around 779.5 and 781.6 eV [28]. The satellite peaks derived from Co 2p 1/2 at 796.9 eV and 794.8 eV might be directed to Co (III) and Co (II) in the tetrahedral sites, respectively [28]. Overall, Co presented a mixed valence of +2 and +3, and the dominance of Co^2+^ here corresponded to its theoretical chemical valence within CMO. The deconvolution of Mn 2p spectra in Figure 3c into three peaks at about 640.1, 641.1, and 643.5 eV also implies the mixed valences of Mn within the catalysts. The binding energies mentioned above were identified as Mn (II), Mn (III), and Mn (IV) in sequence. The Mn 2p 1/2 peak centered near 653.5 eV was possibly caused by Mn (III) at the octahedral site, and the one at 643.5 eV might be Mn^4+^ in MnO_2_ crystal or octahedral Mn^4+^ cation in the spinel structure [29,30,31]. It was interesting to find that the surface ratio of (Mn^2+^ + Mn^3+^)/Mn^4+^ in CMO/Kln was higher than that of CMO. Previous research revealed that the presence of more low-valence Mn might promote the generation of crystallite defects and oxygen vacancies, which helped convert O_2_ into active oxygen species [32]. Thus, the Kln substrate might accelerate the generation of OVs within the catalyst, which was possibly caused by the charge redistribution under the influence of the surrounding Al, Si, and O species. Furthermore, the Kln might also interfere with the crystalline structure of the anchored CMO other than just with its grain size and crystallinity [33]. The O 1s spectra in Figure 3d of the samples could be fitted into three peaks at around 532.6 eV, 531.1eV, and 529.9 eV directing to the physically adsorbed oxygen (O_ads_), hydroxyl oxygen species (O_OH_), and lattice oxygen (O_L_), respectively [26,34,35]. The presence of the Kln substrate significantly increased the content of O_ads_ within CMO/Kln due to its abundant hydroxyl groups, while O_L_ accounted for a lesser amount. In addition, the rich surface-adsorbed water induced by Kln could help enhance the polarity of the catalyst, making it easier to disperse in water, therefore increasing its contact with the organic compounds and PMS [36]. 

The slight shifts frequently observed in Co and Mn spectra of CMO/Kln might be due to the condensation of metal ions with hydroxyls on the surface of minerals. It has also been reported that the ratio of O_ads_/O_L_ could reflect the amount of OVs indirectly since O_ads_ usually originate from the dissociation of adsorbed O_2_ on OVs [37]. The ratio of O_ads_/O_L_ here in CMO was lower than CMO/Kln, which was consistent with the trend of (Mn^2+^+Mn^3+^)/Mn^4+^. This situation implied the plentiful surface OVs in CMO/Kln, indicating that the Kln substrate manifested more surface defects than bare metal oxides [32]. Generally, the XPS results above revealed that natural clay mineral carriers like Kln did not interfere with the chemical compositions of CMO, and the dominant valences of Co and Mn corresponded well with the theoretical calculations. Furthermore, the Kln substrate might help induce OVs into the catalysts, which could enhance their catalytic activity.

**Figure 4:**
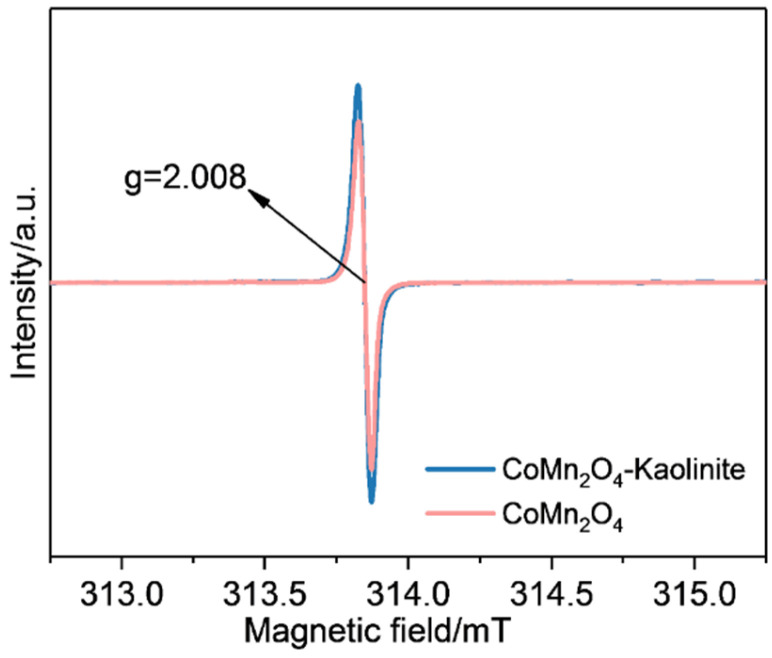
EPR spectra of bare CMO and CMO/Kln.

The existence and differences of OV amount within bare CMO and CMO/Kln were further verified through EPR tests, as shown in Figure 4. The characteristic signal at g = 2.008 that appeared in both bare CMO and CMO/Kln corresponded well with the electrons trapped in the OVs, which demonstrated the existence of OVs [38]. The OVs within the catalysts were widely reported to be beneficial in accelerating the redox cycle of high-valence metal species and the adsorption of PMS, which improved the catalytic efficiency [39]. OVs could also contribute to faster reaction kinetics through an enhanced generation of ^1^O_2_ and additional active sites provided for catalytic activation [40]. The larger integral area of CMO/Kln’s EPR signal revealed its significantly higher content of electrons trapped in the abundant OVs compared with bare CMO. Thus, it could be inferred that the Kln substrate promoted the formation of OVs within the catalysts, which corresponded to the trends in O_ads_/O_L_ and (Mn^2+^+Mn^3+^)/Mn^4+^ between CMO/Kln and bare CMO observed in XPS before. Furthermore, the reduced crystallinity of CMO/Kln compared with bare CMO also indicated its higher OVs content as OVs tended to be formed in atomic structures with higher disorderliness and lower crystallinity [25,41]. Therefore, introducing the Kln carrier was conducive to generating OVs, which provided an alternative to promoting the catalysts’ working efficiency [42].

**Figure 5:**
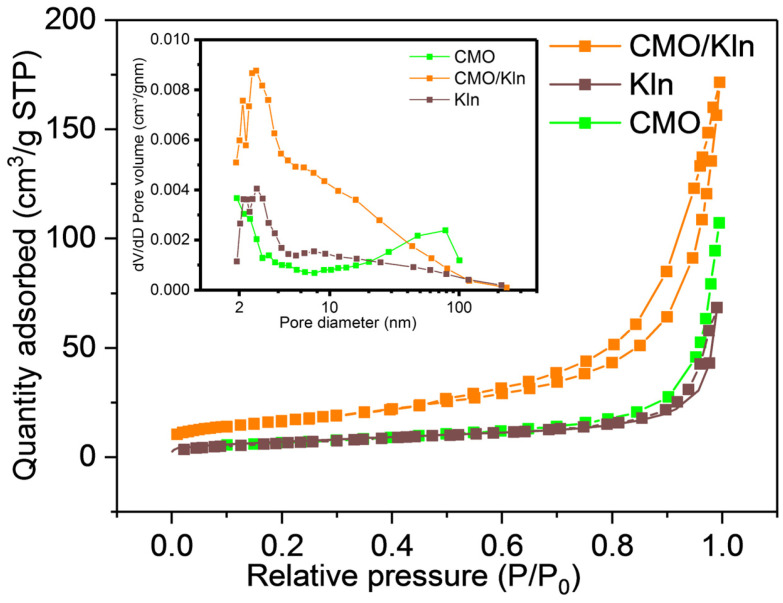
N_2_ adsorption and desorption isotherms and BJH pore size distribution plots for bare CMO, Kln, and CMO/Kln.

The texture of Kln, bare CMO, and CMO/Kln, including specific surface area (SSA) and pore size distributions, were investigated systematically by N_2_ adsorption/desorption isotherm. The isotherm curves in Figure 5 were classified as an IV branch with a typical H3-hysteresis loop, revealing the mesoporous structure of the samples [43]. There was little difference observed in the shapes between bare Kln and CMO/Kln, which might come from the uniform dispersion of CMO nanoparticles under the control of the Kln substrate [44]. The specific surface area (SSA) of CMO/Kln was higher than that of bare CMO and Kln (Table S1) with a slightly reduced pore volume, which might be attributed to the uniform distribution of CMO nanospheres with a smaller grain size and alleviated agglomeration [45]. Additionally, CMO/Kln with higher SSA and pore volume was also beneficial for the adsorption and enrichment of contaminant molecules in the later degradation [46].

### 2.2. Catalytic Performances of the Catalysts through the Activation of PMS

**Figure 6:**
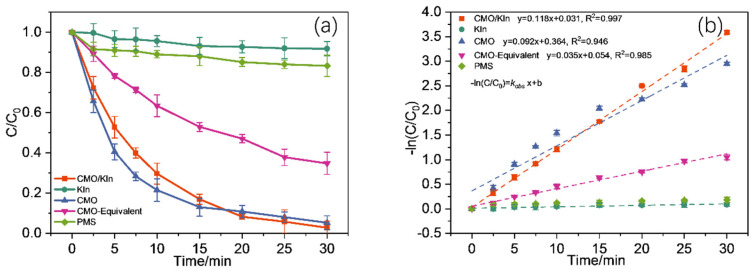
(**a**) Degradation of SMZ in different reaction systems ([CMO+Kln]: 160 mg L^−1^ CMO+240 mg L^−1^ Kln), and (**b**) corresponding pseudo first-order kinetic models. General working conditions: [SMZ] = 20 μmol L^−1^, [PMS] = 0.1 mmol L^−1^, [catalyst] = 400 mg L^−1^, pH = 5.6.

The adsorption of SMZ by bare CMO, Kln, and CMO/Kln was tested separately before the degradation. The results in Figure S1 show that all the mentioned materials could hardly remove SMZ in 40 min without additional assistance although CMO/Kln and Kln showed a slightly better performance than bare CMO, which possibly benefited from the higher SSA and larger pore volume. The results in Figure 6a reveal that sole PMS also showed little impact on the reduction in SMZ, but the simultaneous existence of PMS and CMO-based catalysts significantly enhanced its removal. Among them, CMO/Kln showed the best performance with about 94% of SMZ degraded in 30 min, followed by bare CMO with an unexpected 90% removal rate. The inferior performance of bare CMO might be attributed to its aforementioned serious agglomeration since these lumpy particles with larger grain sizes showed reduced amounts of reactive sites and, thus, less contact with PMS or SMZ. The special advantage of CMO/Kln linked with chemical bonds was verified by another control experiment using ‘Kln + CMO’ as the substitution of the composite catalysts based on their CMO loading ratio. The working efficiency of the simple mixtures in Figure 6a is incomparable with CMO/Kln, indicating that the obvious promotion effect of CMO/Kln was caused by the characterization modification from Kln toward CMO with chemical bonds between them instead of accelerating the decomposition process directly. The data spots of SMZ degradation using PMS activated by the above catalysts in Figure 6b fit well with the pseudo first-order kinetic model, and the K*obs* are provided. The impacts from working parameters such as dosages of SMZ and PMS and working pH on the degradation of SMZ were investigated separately (Detailed in Text S2 and Figures S2 and S3). The optimal working parameters were determined as the general working conditions in this work without special notice. The intermediates generated during the decomposition of SMZ were also detected using LC-MS, and the results are provided in Figure S4. Moreover, compared with bare CMO, more than 25% of SMZ could be mineralized by CMO/Kln (Figure S5). This might also come from its abundant OVs on the surface (Figure 4), which may serve as additional active sites [47]. 

### 2.3. Reaction Mechanism

**Figure 7:**
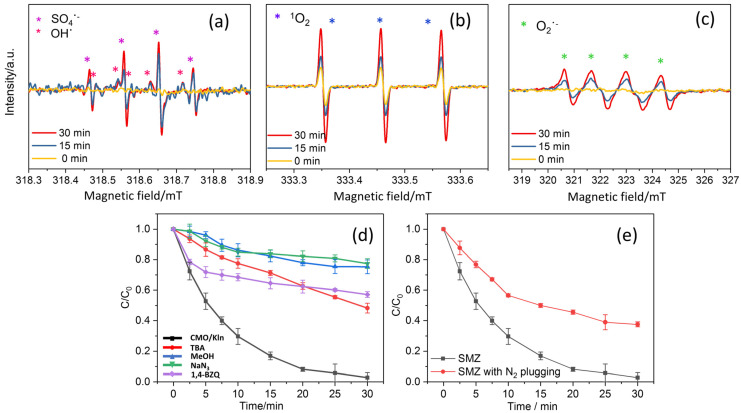
EPR spectra of CMO/Kln: (**a**) SO_4_^·−^ and OH^·^, (**b**) ^1^O_2_, and (**c**) O_2_^·−^. (**d**) Effects of scavengers on SMZ degradation. (**e**) Effect of N_2_ plugging on SMZ degradation. General working conditions: [SMZ] = 20 μM, [PMS] = 0.1 mM, [catalyst] = 400 mg L^−1^, [scavengers] = 10 mM, [spin-trapping reagents] = 0.1 M, pH = 5.6.

EPR tests using DMPO and TEMP as spin-trapping reagents were performed for the identification of ROSs generated in the catalytic PMS activation processes for SMZ degradation. Figure 7a–c shows that the characteristic peaks of the spin adducts, DMPO-OH, DMPO-OOH, and DMPO-SO_4_, were detected just minutes after the addition of PMS, confirming the co-existence of SO_4_^·−^, OH,^·^ and O_2_^·−^ during PMS activation [48]. However, the triplet peaks of TEMPO from ^1^O_2_ appeared simultaneously with the addition of PMS, indicating the possible existence of its precursor with sole catalysts in the solution. The intensity of TEMPO surged dramatically several minutes after the addition of PMS, which might indicate its participation in ^1^O_2_ formation. Therefore, the origin of ^1^O_2_ might not be restricted to PMS but also have other sources. The EPR signals of the four ROSs maintained a strong presence for at least 30 min after initiation, indicating their continuous generation during the degradation, which guaranteed the sustainable oxidation of SMZ. 

Traditional trapping experiments were also performed separately with radical scavengers, including MeOH, TBA, NaN_3_, and 1, 4-BZQ, to further confirm their participation in SMZ oxidation. The reaction rate constants between the scavengers and their target ROSs are illustrated in Table S2. Figure 7d reveals that the addition of MeOH strongly inhibited the removal of SMZ using PMS activated by either CMO or CMO/Kln, while only marginal reductions were observed with TBA. These results confirmed the involvement of SO_4_^·−^ and OH^·^ in SMZ degradation, with SO_4_^·−^ being the dominant one. Moreover, trapping experiments using NaN_3_, and 1, 4-BZQ targeted at ^1^O_2_ and O_2_^·−^ with obvious retardation further implied their unignorable contributions to the degradation. Thus, all four ROSs mentioned were successfully formed during PMS activated by either CMO or CMO/Kln and then participated in SMZ degradation to a different extent.

According to the XPS analysis on the fresh and recycled CMO/Kln, the valence of Co and Mn both changed (Figure S6). A decrease in Co^2+^ accompanied by increased Co^3+^ was observed on CMO/Kln, indicating the oxidation of Co^2+^ into Co^3+^ by electron transfer toward PMS, from which SO_4_^·−^ could be generated. Afterward, the reduction of Co^3+^ back to Co^2+^ with HSO_5_^−^ contributed to the formation of SO_5_^·−^ or OH^·^ (Equations (1)–(3)) [49].

Co^2+^ + HSO_5_^−^ → Co^3+^ + SO_4_^·−^ + OH^−^ (1)

Co^3+^ + HSO_5_^−^ → Co^2+^ + SO_5_^·−^+ H^+^ (2)

Co^3+^ + HSO_5_^−^ → Co^2+^ + SO_4_^2−^ + OH^·^ (3)

Similar variation was also observed in Mn on CMO/Kln as the proportion of Mn^2+^ increased with a decrease in Mn^3+^ after the reactions. This implies that a similar electron transfer happened between Mn^2+^ and Mn^3+^ (Equations (4) and (5)) [50]. The redox reaction between Mn^4+^ and Mn^3+^ was also feasible (Equations (6)–(9)).

Mn^3+^ + HSO_5_^−^ → Mn^2+^ + SO_5_^·−^ + H^+^ (4)

Mn^2+^ + HSO_5_^−^ → Mn^3+^ + SO_4_^·−^ + OH^−^ (5)

Mn^3+^ + HSO_5_^−^ → Mn^2+^ + SO_4_^2−^ + OH^·^ (6)

Mn^3+^ + HSO_5_^−^ → Mn^4+^ + SO_4_^·−^ + OH^−^ (7)

Mn^4+^ + HSO_5_^−^ → Mn^3+^ + SO_5_^·−^ + H^+^ (8)

Mn^4+^ + HSO_5_^−^ → Mn^3+^ + SO_4_^2−^ + OH^·^ (9)

Furthermore, due to a higher standard of reduction potential of Co^3+^/Co^2+^ (1.81 V) compared with Mn^3+^/Mn^2+^ (1.51 V) and Mn^4+^/Mn^3+^ (0.15 V), the reduction of Co^3+^ back to Co^2+^ by Mn^3+^/Mn^2+^ and Mn^4+^/Mn^3+^ was thermodynamically favorable [50] The O 1s spectra showed that the content of O_L_ within CMO/Kln after the use remained almost unchanged, indicating that it was less active than Co and Mn. This phenomenon was consistent with some previous research findings revealing that catalysts with higher O_L_ tended to follow the lattice oxygen oxidation mechanism instead of those with less O_L_ [51].

The experimental results obtained under N_2_ purging, shown in Figure 7e, imply that without dissolved oxygen (DO), the removal rate of SMZ using PMS activated by CMO/Kln was reduced by about 60%. This revealed the important role DO played in the oxidation process. Some previous research studies reported that DO played an important part in ROS generation, i.e., DO could act as a precursor of ^1^O_2_ at the early stage of the reaction or as an electron from OVs to yield the generation of O_2_^·−^, which further transformed into ^1^O_2_ [52,53]. The previous EPR results in Figure 4 confirm the existence of OVs within CMO/Kln, implying its feasibility in participating in the generation of ROS. Thus, it was reasonable to assume the importance of OVs and DO in the generation of O_2_^·−^ and ^1^O_2_ from the retardation of SMZ degradation under anaerobic environment. The OVs on the surface of catalysts with abundant single electrons could contribute to the interfacial electron transfer during PMS activation. It has been reported that the oxygen-deficient OVs on the surface of the catalysts usually show strong Lewis acidity. Thus, PMS (HSO_5_^−^) tended to be adsorbed on OVs with low oxygen coordination, which could later be decomposed and released into the solution with the formation of active oxygen species (O*) (Equation (10)) [54]. Furthermore, due to the abundant surface hydroxyl groups on the Kln substrate, the synergistic effects between Kln and rich OVs might also assist or participate in PMS activation. Moreover, lattice oxygen species located near surface OVs might also be released and turn directly into O*, with the generation of ^1^O_2_ from the coupling of O* and HSO_5_^−^ (Equation (11)) [53]. In addition, relevant studies also reported that dissolved oxygen (DO) was able to accept an electron from OVs to yield O_2_^·−^ (Equation (13)), which could be converted into ^1^O_2_ via Equation (12) [55,56]. The participation of DO was consistent with the retardation of SMZ degradation under N_2_ purging (Figure 7e).

Ov → O*(10)

O* + HSO_5_^−^ → ^1^O_2_ + HSO_4_^−^ (11)

O_2_^·−^ + OH^·^ → ^1^O_2_ + OH^−^ (12)

O_2_ + e^−^ → O_2_^·−^ (13)

### 2.4. Recyclability and Stability of the Catalysts

**Figure 8:**
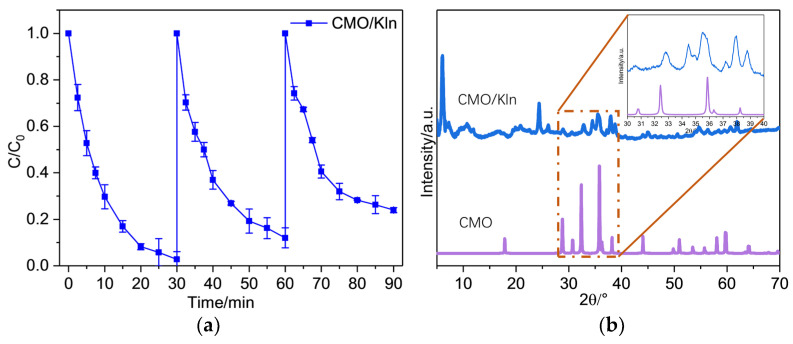
(**a**) SMZ degradation with CMO/Kln after three recycle rounds. (**b**) XRD pattern of recycled CMO/Kln compared with fresh CMO. General working conditions: [SMZ] = 20 μM, [PMS] = 0.1 mM, [catalyst] = 400 mg L^−1^, pH = 5.6.

The durability of CMO/Kln was investigated by continuous degradation experiments, and the leakage of Co and Mn from them was determined by ICP-OES at the end of each working round. Figure 8a implies that CMO/Kln maintained a satisfactory catalytic performance with little retardation after being reused three times, with about 75% of SMZ removed in the last turn. 

The dissolution of Co and Mn from CMO/Kln with an additional mineral carrier was obviously alleviated compared with the bare ones (Figure S7a,b). The concentration of leached Co and Mn from CMO/Kln was less than 0.1 ppm, which was less than half of that from bare CMO. Besides, this leakage concentration of Mn and Co met the limits of Chinese environmental quality standards for surface water (Mn < 1.0 ppm, Co < 0.1 ppm) [57]. Furthermore, the dosages of CMO and CMO/Kln were the same in the recycle tests, indicating that CMO/Kln contained only 40% of bare CMO; however, the metal loss was still more than 40% lower than in bare CMO (Figure S7a,b). Thus, the addition of mineral carriers could greatly reduce metal leaching and effectively promote the stability of the catalysts, which might be due to their special porous structures and functional hydroxyl groups [58,59]. 

Homogeneous catalytic degradation was performed to investigate whether the leached Co and Mn would help activate PMS in addition to the heterogeneous catalysts. Herein, the precursors of the catalysts during synthesis, Co(NO_3_)_2_ and Mn(NO_3_)_2_, were adopted as the sources of metal ions. The substitution of Mn^2+^ for Mn^3+^ was reasonable here as dissolved Mn from manganese oxides or hydroxides through redox reactions mainly showed +2 valence in the solution, and Mn^3+^ was unstable and eager to convert into Mn^2+^ through the disproportionation reaction in the aqueous environment [60]. The dosages of Co(NO_3_)_2_ and Mn(NO_3_)_2_ were separately set as 0.1 ppm and 1.0 ppm to correspond with the highest leaching concentration from CMO/Kln. Figure S7c clearly shows that about 30% of SMZ was removed by Co^2+^ in 30 min while little change was observed with Mn^2+^, confirming that homogeneous PMS activation by Co^2+^ was feasible. However, it could be concluded that the contributions from the leached ions were far less effective and the catalyst still played a dominant role. Furthermore, the unavoidable metal leakage of CMO/Kln also contributed to a reduction in degradation efficiency, simultaneously with blocking or covering the reaction sites by molecules like SMZ and its degradation intermediates. 

Since the XPS spectra could only verify the surface chemical composition without monitoring the structural changes, the XRD test was performed again on the recycled CMO/Kln catalysts. Figure 8b implies that little changes, including some possible peak shifts, were found on the XRD patterns of the recycled CMO/Kln, except for a slight reduction in its intensity. This phenomenon revealed the stable crystalline structure of CMO/Kln after being reused three times, which confirmed its high chemical stability with a potential for a long run. The above results implied that the Kln substrate was beneficial for inhibiting metal dissolution from CMO, and CMO/Kln with reduced crystallinity and a smaller grain size than CMO also maintained structural stability after 3 times of reuse [61].

### 2.5. Degradation Performance of CMO/Kln on Some Other Pharmaceuticals 

The extensive and universal usage of CMO/Kln was further tested on three other frequently detected pharmaceuticals in the aquatic environment, tetracycline (TC), ofloxacin (OFX), and carbamazepine (CBZ). The degradation was performed at normal pH (without special adjustment) for 40 min, and the results in Figure S8 reveal the effectiveness of CMO/Kln for these pharmaceuticals. Thus, CMO/Kln could still decompose PMS into plenty of ROS to oxidize these organics, and the slightly inferior performances here were possibly caused by the unsuitable working parameters applied directly from the SMZ degradation. 

## 3. Materials and Methods

### 3.1. Chemical Reagents

The Kln used in this research was from Shanxi, Linfen, China. Its chemical components were determined by XRF and are provided in Table S3. All the chemical reagents used in this research were used as received without any further purification (detailed in Text S3). All the experiments, including solution preparation and batch experiments, were carried out with Milliq water at room temperature (25 °C). 

### 3.2. Synthesis and Characterizations of the Catalysts

**Scheme 1:**
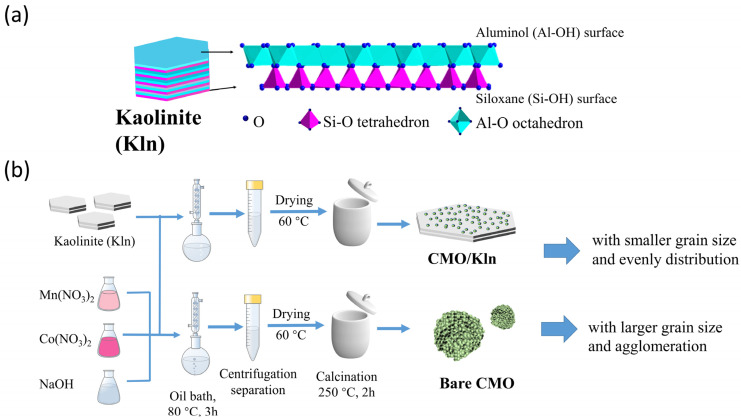
The crystalline structure of planar kaolinite (Kln) (**a**) and synthesis route of bare CMO and CMO/Kln (**b**).

The catalysts were fabricated by the co-precipitation method and were named CMO or CMO/Kln based on their chemical composition (briefed in Scheme 1 and detailed in Text S4). The obtained catalysts were characterized systematically for crystal structure, morphology, surface composition, specific surface area, pore distribution, and OV content with XRD, FESEM, HAADF-STEM, XPS, BET, and EPR analysis. More information about the characterization instruments and working parameters was provided in detail in Text S5.

### 3.3. Batch Experiments and Analytical Methods

The catalytic performances of CMO and CMO/Kln were evaluated by the degradation of SMZ through PMS activation. The catalysts were added to the working solution for another 15 min to obtain the adsorption–desorption equilibrium. The reaction was then initiated by the addition of predetermined dosages of PMS. At certain time intervals, 1 mL of the suspension was taken out by a syringe and filtered through a 0.45 μm PES membrane plate and then quickly quenched with 0.1 M Na_2_S_2_O_3_ to terminate the reaction. 

The residue concentration of SMZ and some other pharmaceuticals adopted in this work was determined by high-performance liquid chromatography (HPLC). The reactive oxygen species (ROS) generated from PMS activation were determined by electron paramagnetic resonance (EPR). The metal leakage from the catalysts during the degradation was analyzed by inductively coupled plasma-optical emission spectroscopy (ICP-OES) after HNO_3_ digestion. The mineralization efficiency of SMZ degradation was measured by the reduction in total organic carbon (TOC). Detailed information on the instruments and their working parameters is provided in Text S6.

## 4. Conclusions

In this research, bare CMO and CMO/Kln with natural kaolinite substrate were fabricated for PMS activation to remove SMZ from water. The results confirmed the superior catalytic activity of CMO/Kln with lower metal consumption for fabrication and less metal leakage during its operation. The CMO/Kln using less metal but having a comparable performance with bare CMO might be due to the smaller CMO grain size loaded on the Kln substrate with better and more even dispersion, which provided more active sites for PMS activation. It was reasonable to conclude that the porous Kln mineral substrate helped alleviate metal leaching during reaction processes, thus creating the composite catalyst with higher chemical and structural stability. The effective removal of three other pharmaceuticals using PMS activated by CMO/Kln further proved its universal applicability. This work revealed that it was feasible to use Kln, an abundant natural clay mineral with lamellar morphology and rich surface hydroxyl groups, as an effective substrate for CMO particles for PMS activation with special advantages like easier loading, controlled grain sizes, and higher structural stability, which alleviated metal leaching and, thus, avoided secondary pollution to a certain extent. 

The main working ROS in the oxidation process were SO_4_^·−^, OH^·^, ^·^O_2_^−^ and ^1^O_2_, which were mainly achieved through electron transfer, and the feasible redox reactions between Co and Mn guaranteed the continuous generation of SO_4_^·−^ and OH^·^. The mineralization rate at more than 25% obtained from the TOC test revealed that near-complete decomposition of SMZ into inorganics could be achieved after being treated with this oxidation process. Generally, this work provides a novel reference to the feasible application of natural minerals as effective catalyst carriers while obviously promoting the catalytic ability for metal-based catalysts in wastewater treatment.

## Data Availability

The data presented in this study are available on request from the corresponding author.

## Supplementary Materials

The following supporting information can be downloaded at: https://www.mdpi.com/article/10.3390/molecules29163825/s1. Text. S1 Scherrer equation for the calculation of average grain size; Text. S2 Effects of working parameters: dosage of SMZ, PMS, and working pH; Text. S3 Chemical reagents adopted in this work; Text. S4 Preparation of the catalysts; Text. S5 Characterization of the catalysts; Text. S6 Analytical methods; Table S1 Specific surface area and pore volume of bare CMO, Kln, and CMO/Kln.; Table S2 Target ROSs and the corresponding scavengers; Table S3 Chemical composition of kaolinite (Kln) tested by XRF; Figure S1 Adsorption of SMZ by CMO, Kln, and CMO/Kln; Figure S2. Degradation performances of CMO/Kln; Figure S3. Zeta potential of CMO/Kln; Figure S4. Intermediates during SMZ degradation using PMS activated by CMO/Kln; Figure S5. TOC reduction of SMZ, OFX, CBZ and TC.; Figure S6. XPS spectra of recycled CMO/Kln, and its variations with the fresh ones.; Figure S7. ICP-OES results of CMO and CMO/Kln in recycle experiments and homogeneous degradation of SMZ using Co^2+^ and Mn^2+;^ Figure S8. Catalytic degradation performances of three other pharmaceuticals using PMS activated by CMO/Kln.
