# Supplementary material for: Introducing and Boosting Oxygen Vacancies within CoMn2O4 by Loading on Planar Clay Minerals for Efficient Peroxymonosulfate Activation
Source: Molecules. 2024 Aug 12;29(16):3825. doi: 10.3390/molecules29163825 (PMC11357143; doi:10.3390/molecules29163825)
Supplement: Supplementary file 1 [file molecules-29-03825-s001.zip › molecules-3134090-supplementary.pdf]

# Scheme 2. O4 by loading on planar clay minerals for efficient peroxymonosulfate activation.

**Xue Yang [1], Xiao Yao [1], * and Yinyuan Qiu [2, 3],***

1 School of Ecological Environment and Urban Construction, Fujian University of Technology, Fuzhou 350118, China
2 Fujian Special Equipment Inspection and Research Institute, Fuzhou 350008, China
3 School of Mechanical and Automotive Engineering, Fujian University of Technology, Fuzhou 350118, China
* Correspondence: 18041778r@connect.polyu.hk (Xiao Yao); yinyuan102@fjut.com (Yinyuan Qiu)

**Text. S1 Scherrer equation for the calculation of average grain size**
**Text. S2 Effects of working parameters: dosage of SMZ, PMS, and working pH**
**Text. S3 Chemical reagents adopted in this work**
**Text. S4 Preparation of the catalysts**
**Text. S5 Characterization of the catalysts**
**Text. S6 Analytical methods**

**Table S1 Specific surface area and pore volume of bare CMO, Kln, and CMO/Kln.**
**Table S2 Target ROSs and the corresponding scavengers**
**Table S3 Chemical composition of kaolinite (Kln) tested by XRF**

**Figure S1 Adsorption of SMZ by CMO, Kln, and CMO/Kln**
**Figure S2. Degradation performances of CMO/Kln**
**Figure S3. Zeta potential of CMO/Kln**
**Figure S4. Intermediates during SMZ degradation using PMS activated by CMO/Kln**
**Figure S5. TOC reduction of SMZ, OFX, CBZ and TC.**
**Figure S6. XPS spectra of recycled CMO/Kln, and its variations with the fresh ones.**
**Figure S7. ICP-OES results of CMO and CMO/Kln in recycle experiments and homogeneous degradation of SMZ using $Co^{2+}$ and $Mn^{2+}$**
**Figure S8. Catalytic degradation performances of three other pharmaceuticals using PMS activated by CMO/Kln.**

## Text. S1 Scherrer equation for the calculation of average grain size

The average crystal size of CMO and CMO loaded on Kln were calculated through the Scherrer equation separately considering their most intense peaks.

$$D=K\lambda/(\beta\cos\theta),$$

where D is the nanoparticles crystalline size, K represents the Scherrer constant, i.e., the particle shape factor (0.9 here), λ denotes the X-ray wavelength (0.15406 nm here), and β denotes the full width at half maximum at (*hkl*) reflection.
Herein, the crystalline size of the nanoparticles was evaluated using the most intense Braggs peak of CMO located at 2θ=36.89°, corresponding to (211) reflection. Therefore, the grain size of both CMO and CMO loaded on Kln substrate could be calculated at about 26 and 12 nm, respectively.

## Text. S2 Effects of working parameters: dosage of SMZ, PMS, and working pH

The effects from SMZ and PMS dosages were indicated in Figure S2. The significantly lower degradation of SMZ with the increasing of its concentration could easily be explained by the fact that limited PMS dosage restricted the further generation of ROSs for SMZ oxidation. Similar explanation could also be used on the effect of PMS concentrations' variation on SMZ degradation, since higher the PMS concentration, larger amount of ROSs could be generated.
In order to determine the impact of working pH, the pHpzc of CMO/Kln was tested. The pHpzc of Kln was reported at around 2.8-2.9, but after anchoring CMO, the pHpzc changed significantly, with CMO/Kln at around 5.5 as indicated in Figure S3. Figure S2 revealed that the optimal working pH to remove SMZ through PMS activation by CMO/Kln was 5.6, with more SMZ removed. However, when working pH varies, the performance of the composite catalyst changed somehow. Figure S5 indicated that degradation efficiency of CMO/Kln was retarded under both acidic and alkaline environments like pH=4.2 and pH=8.6, and the whole reaction was strongly suppressed at pH=10.1.

Except this, another reason might be related to the acid dissociation constants ($pK_{a1}$=1.49, $pK_{a2}$ =5.41) of SMZ. The two acid dissociation constants could be directed to two ionizable functional groups, amino group ($N^1$) and amide nitrogen atom. Based on the acid dissociation constants of SMZ, it could be recognized as zwitterionic while CMO/Kln were slightly negatively charged at pH=5.6, therefore, it could be sorbed by the catalysts through cation exchange and other surface complexation such as hydrogen bonding. When pH exceeded the range between the two $pK_a$, the electrostatic repulsion between the catalysts and SMZ interfered the adsorption and degradation efficiency.

**Text. S3 Chemical reagents adopted in this work**

The synthetic raw materials for the catalyst included Cobalt (II) nitrate ($Co(NO_3)_2 \cdot 6H_2O$), Manganese (II) nitrate ($Mn(NO_3)_2 \cdot 6H_2O$), and Sodium hydroxide (NaOH), which were purchased from Macklin Inc., China. Other chemical reagents included were listed below. Peroxymonosulfate (PMS, $KHSO_5 \cdot 0.5KHSO_4 \cdot 0.5K_2SO_4$) was purchased from Acros Organics. Quenchers for the traditional trapping experiments includes Tert-butyl alcohol (TBA, provided by Sigma-Aldrich), 1,4-benzoquinone (1,4-BZQ, provided by Sinopharm Chemical Reagent Co., Ltd), sodium azide ($NaN_3$, provided by Sigma-Aldrich) and Methanol (MeOH, provided by Anaqua Global International Inc. Limited). $H_2SO_4$ was purchased from RCI Labscan Limited. The mobile phases for High-Performance Liquid Chromatograph (HPLC) analysis were: Acetonitrile from DUKSAN Reagents; MeOH and Oxalic acid from Sigma-Aldrich. In addition, all the solvents adopted in the HPLC analysis were HPLC grade. The spin trapping agents for EPR analysis were 5, 5-dimethyl-1-pyrrolidine N-oxide (DMPO) and 2,2,6,6-tetramethylpiperidine (TEMP). All the chemicals were of analytical purity (AR) or ACS grade and were used directly without further purification. Water from Millipore Waters Milli-Q water purification system was used throughout the whole experiments. Target compounds including Ofloxacin (OFX), Carbamazepine (CBZ), Sulfamethoxazole (SMZ) and Tetracycline (TC) were purchased from Sigma-Aldrich.

**Text. S4 Preparation of the catalysts**

Typically, 2g kaolinite (Kln) was dispersed in 200 ml Milliq water and kept stirring for 6h. Then, based on the predetermined weight ratio (40% here), certain amount of Cobalt (II) nitrate ($Co(NO_3)_2 \cdot 6H_2O$) and Manganese (II) nitrate ($Mn(NO_3)_2 \cdot 6H_2O$) were resolved in the natural mineral suspension spontaneously for another 2 hours to make them evenly distributed, followed by the addition of overdosed 4M NaOH. Then the whole fabrication system was heated with oil bath at 80 °C for 3 hours under continuous stirring. During this process, the particles would generate gradually. After the oil bath was removed, the reactor was cooled to room temperature (about 20 °C) naturally. The precipitate was separated using centrifugation at small amount each time and dried at 60 °C for 6 hours until it was completely dry. The obtained dried powder was moved into a muffle furnace and calcinated at 250 °C for 2 hours. Then the obtained powder was grounded and named as CMO/Kln. The bare $CoMn_2O_4$ (CMO) was prepared through the same process without the addition of Kln.

**Text. S5 Characterization of the catalysts**

The crystal structures of the catalysts were determined by D8 ADVANCE X-ray diffractometer (XRD, Bruker, Germany) with Cu Kα as the radiation source (λ = 0.15418 nm). The surface composition and chemical valence of the catalysts were analyzed by X-ray photoelectron spectroscopy (XPS) (ESCALAB 250, Thermo Fisher Scientific, USA), using Al Kα (hv=1486.8 e V) as the excitation light source and C 1s = 284.4 eV as the calibration to obtain the binding energy of each element species on the catalyst surface. The surface morphology was examined by field emission scanning electron microscope (FESEM, Zeiss, USA). The morphology of the powder catalyst samples were analyzed by a FEI High-angle annular dark-field scanning transmission electron microscopy (HAADF-STEM, Talos F200S, USA) equipped with an energy-dispersive X-ray spectroscopy (EDS) at an acceleration voltage of 200 kV. The electron paramagnetic resonance (EPR, JES-FA-300, JOEL, Japan) was used to elucidate the OVs within the catalysts. The Brunauer–Emmett–Teller (BET) analysis was performed by Accelerated Surface Area and Porosity System (Micromeritics) to obtain the specific surface areas and distribution of pore sizes of the complex catalysts after degassing. The zeta potential of the catalysts was measured by a Zetasizer produced by Malvern.

**Text. S6 Analytical methods**

The residue concentration of SMZ and other pharmaceuticals was measured by a Waters 2696 high-performance liquid chromatography (HPLC) system equipped with C18 column and 2487 UV detector. Other working parameters were provided below.

| Organic compound | Mobile phase A | Mobile phase B | Mobile phase C | Wavelength (nm) |
|---|---|---|---|---|
| Ofloxacin | 75% water with 0.1% formic acid | 25% acetonitrile | 0 | 294 |
| Carbamazepine | 40% water | 60% acetonitrile | 0 | 286 |
| Sulfamethoxazole | 40% water | 60% acetonitrile | 0 | 288 |
| Tetracycline | 72% 0.1M Oxalic acid | 20% acetonitrile | 8% methanol | 357 |

The EPR spectroscopy (EPR, JES-FA-300, JOEL, Japan) were performed to determine the ROS generated during catalytic degradation processes using 5,5-dimethyl-1-pyrroline-N-oxide (DMPO) and 2,2,6,6-tetramethyl-4-piperidone (TEMP) as spin reagents.
The concentration of leached metal ions from the catalysts during the degradation was analyzed by inductively coupled plasma-optical emission spectroscopy (ICP-OES, SpectroBlue, Germany) after digesting with a 68% (w/w) $HNO_3$.
The total organic carbon (TOC) reduction was tested by a Shimadzu TOC-5000A analyzer equipped with an ASI-5000A autosampler (Shimadzu, Japan) to determine the mineralization efficiency.

The degradation intermediates of SMZ were determined by Thermo Scientific Orbitrap MS coupled with LC under ESI+ mode. The detected mass rage was 50-2,000 (m/z). The mass accuracy was less than 3 ppm RMS using external calibration. The resolution was 500,000 FWHM at m/z of 200. Acetonitrile and 0.1% formic acid were used as the mobile phases indicated as A and B, respectively. A linear gradient progressed from 10% A (0–2 min) to 70% A in 2–15 min, maintained at 70% A for 3 min, and finally went back to the initial mobile-phase. Nitrogen was employed as both drying and nebulizer gas. Formic acid (0.1%) and isopropyl alcohol were used as the wash solvent.

| ESI mode | Flow rate | Injection volume | Column temperature |
|---|---|---|---|
| positive | 0.25 mL/min | 10 μL | 30 °C |

**Table S1.** Specific surface area and pore volume of bare CMO, Kln, and CMO/Kln.

| **Samples** | **SSA ($m^2g^{-1}$)** | **Pore volume ($cm^3g^{-1}$)** |
|---|---|---|
| CMO | 25.88 | 0.18 |
| Kln | 27.73 | 0.15 |
| CMO/Kln | 28.99 | 0.11 |

**Table S2.** Target ROSs and the corresponding scavengers.

| **Scavenger** | **Target ROSs** | **Reaction rate $k_{obs}$** |
|---|---|---|
| Tert-butyl alcohol (TBA) | $OH^{\bullet-}$ | $k_{OH\bullet-} = (3.8–7.6) \times 10^8\ M^{-1} \cdot s^{-1}$<br>$k_{SO4\bullet-} = (4.0–9.1) \times 10^5\ M^{-1} \cdot s^{-1}$ |
| Methanol (MeOH) | $OH^{\bullet-}$, $SO_4^{\bullet-}$ | $k_{OH\bullet-} = (1.2–2.8) \times 10^9\ M^{-1} \cdot s^{-1}$<br>$k_{SO4\bullet-} = (1.6–7.7) \times 10^7\ M^{-1} \cdot s^{-1}$ |
| Sodium azide ($NaN_3$) | $^1O_2$ | $k = 2.2\times10^9\ M^{-1} \cdot s^{-1}$ |
| 1,4-Benzoquinone (BZQ) | $O_2^{\bullet-}$ | $k = 2.9 \times 10^9\ M^{-1} \cdot s^{-1}$ |

**Table S3.** Chemical composition of kaolinite (Kln) tested by XRF.

| **No.** | **Component** | **Unit mass %** |
|---|---|---|
| 1 | MgO | 0.285 |
| 2 | $Al_2O_3$ | 42.5 |
| 3 | $SiO_2$ | 52.7 |
| 4 | $P_2O_5$ | 0.69 |
| 5 | $K_2O$ | 2.09 |
| 6 | CaO | 0.13 |
| 7 | $TiO_2$ | 0.467 |
| 8 | MnO | 0.0121 |
| 9 | $Fe_2O_3$ | 0.565 |
| 10 | CuO | 0.0321 |
| 11 | SrO | 0.0158 |
| 12 | PbO | 0.511 |

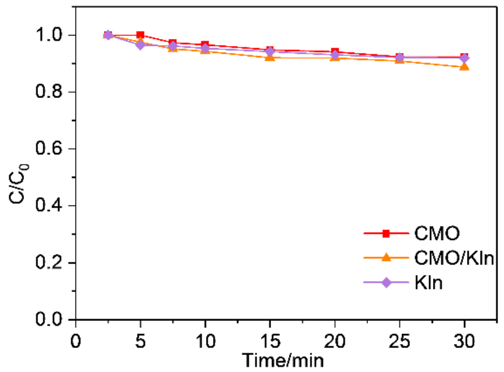


**Figure S1.** Adsorption of SMZ by CMO, Kln, and CMO/Kln. General working conditions: [SMZ]= 20 μM, [PMS]= 0.1 mM, [catalyst]= 400mg $L^{-1}$, pH=5.6

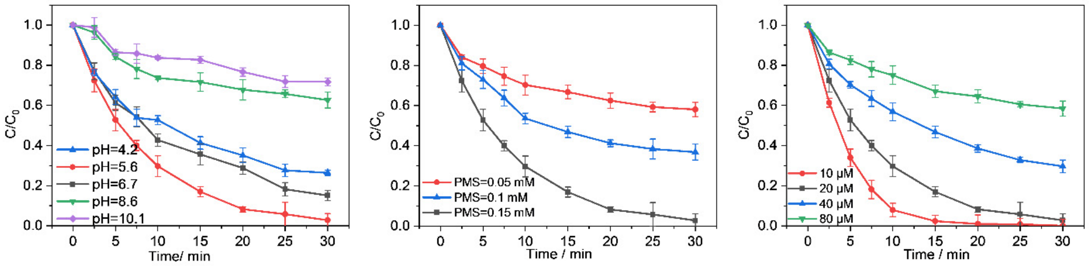


**Figure S2.** Degradation performances of CMO/Kln. (a) SMZ concentration, (b) working pH, and (c). PMS concentration. General working conditions: [SMZ]= 20 μM, [PMS]= 0.1 mM, [catalyst]= 400mg $L^{-1}$, pH=5.6.

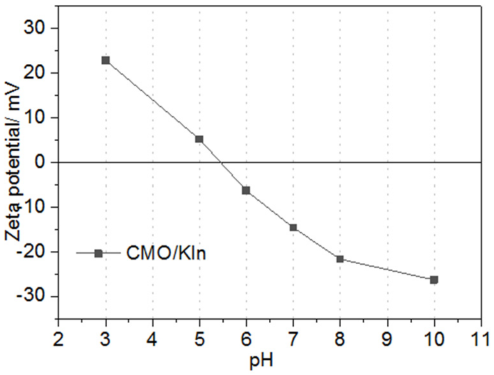


**Figure S3.** Zeta potential of CMO/Kln.

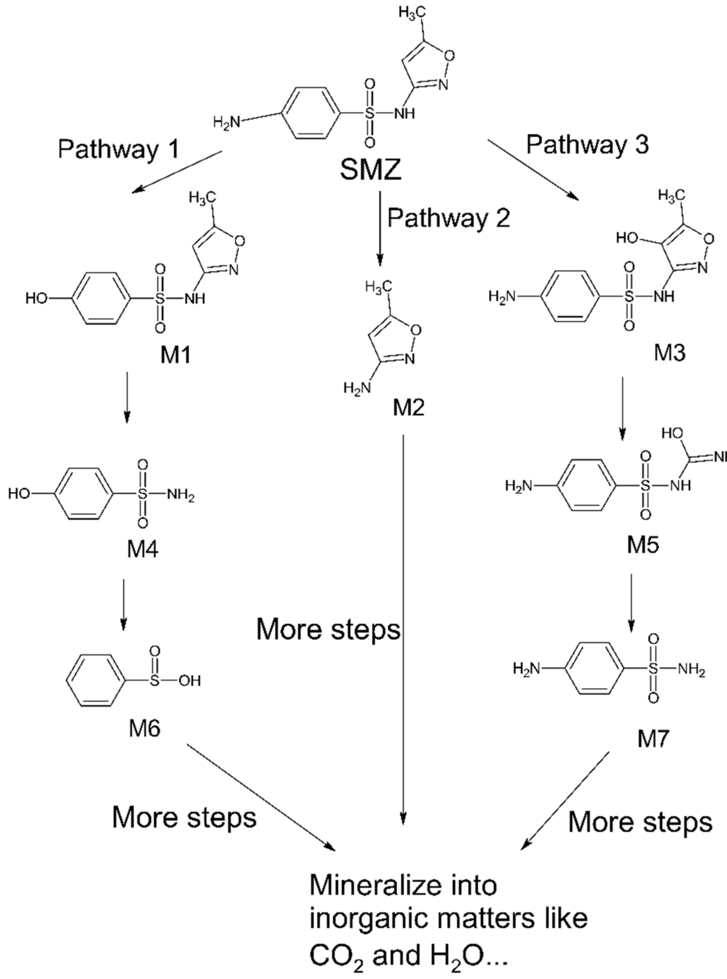


**Figure S4.** Intermediates during SMZ degradation using PMS activated by CMO and CMO/Kln.

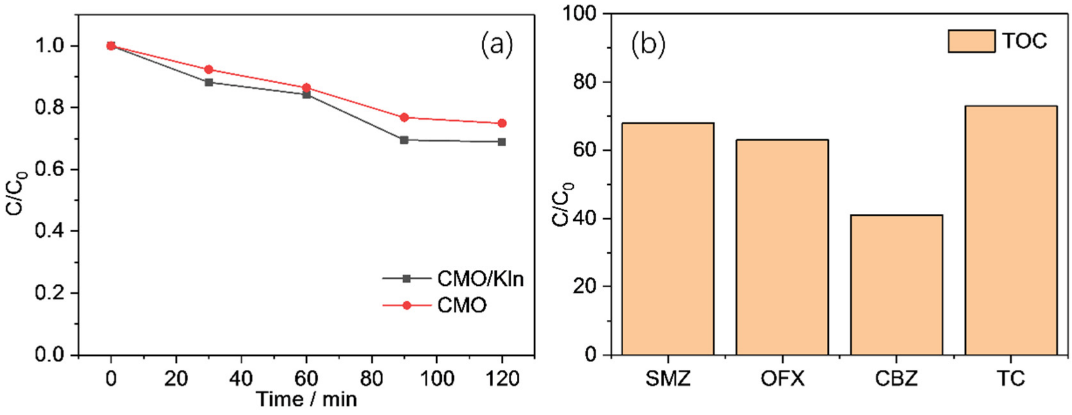


**Figure S5.** TOC reduction during SMZ degradation using PMS activated by CMO and CMO/Kln (a), and TOC reduction of other pharmaceuticals using CMO/Kln.

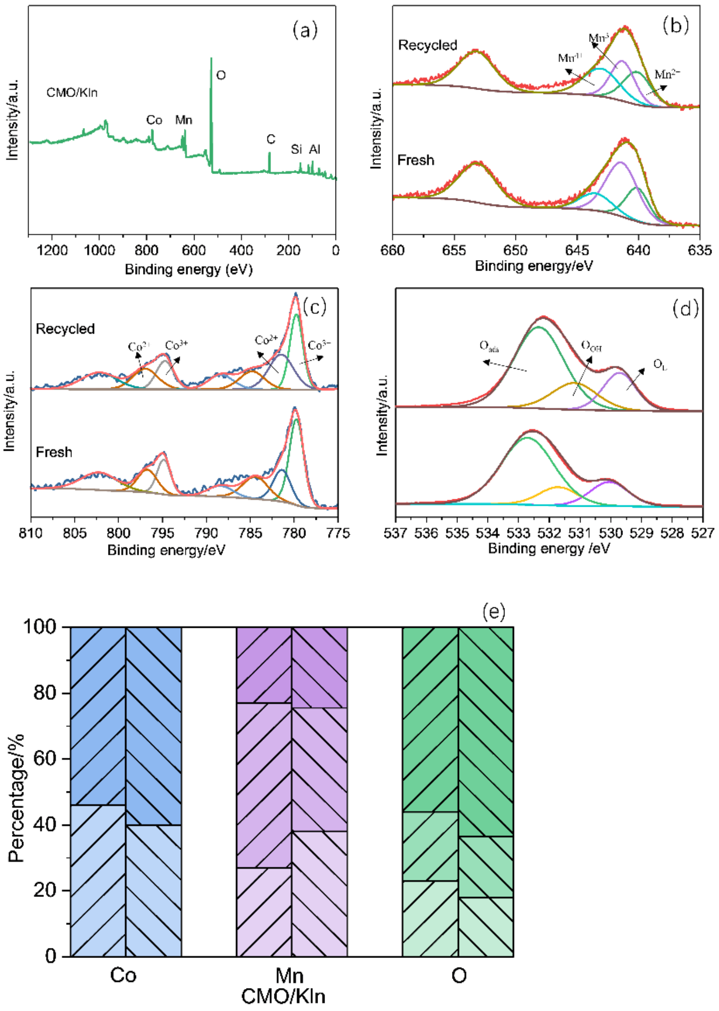


**Figure S6.** XPS spectra of recycled CMO/Kln, and its variations with the fresh ones.

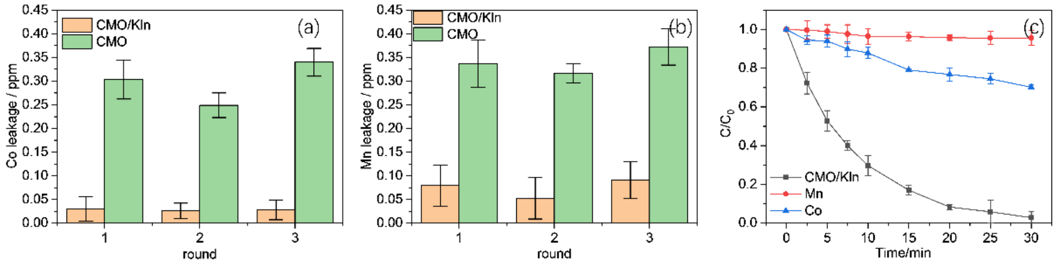


**Figure S7.** ICP-OES results of CMO and CMO/Kln in recycle experiments (a-b), and homogeneous degradation of SMZ using $Co^{2+}$ and $Mn^{2+}$ (c).

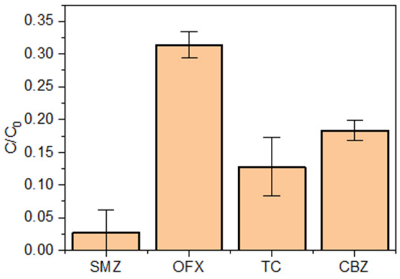

**Figure S8.** Catalytic degradation performances of three other pharmaceuticals using PMS activated by CMO/Kln. General working conditions: [pharmaceuticals]=20 μM, [PMS]= 0.1 mM, [catalyst]= 400mg $L^{-1}$, pH=5.6.
